# Prevalence of depression and associated factors among Somali refugee at melkadida camp, southeast Ethiopia: a cross-sectional study

**DOI:** 10.1186/s12888-015-0539-1

**Published:** 2015-07-24

**Authors:** Fetuma Feyera, Getnet Mihretie, Asres Bedaso, Dereje Gedle, Gemechu Kumera

**Affiliations:** 1 Lecturer, department of Nursing, Debra Markos University college of Medicine and Health Science, Debra Markos, Ethiopia; 2 Lecturer, department of psychiatry, University of Gondar, college of Medicine and Health Science, Gondar, Ethiopia; 3 Lecturer, department of Nursing, Arbaminchi University college of Medicine and Health Science, Arbaminchi, Ethiopia; 4 Lecturer, department of Public health, Debra Markos University college of Medicine and Health Science, Debra Markos, Ethiopia

**Keywords:** Depression, Refugee

## Abstract

**Background:**

Psychological distress, psychosomatic complaints and clinical mental disorders such as depression and post-traumatic stress disorder are highly prevalent among refugees than other populations. Even though there were several studies done on mental health of refugees globally, there is very few in Ethiopia regarding the mental health of these vulnerable populations. Thus we aimed at determining the prevalence of depression and identifying determinants of depression among refugees.

**Methods:**

A community based cross-sectional multistage survey with 847 adult refugees was conducted in May 2014 at Melkadida camp, Southeast Ethiopia. Data were collected by face to face interviews on socio demographic by using structured questionnaire, level of exposure to trauma by Harvard Trauma Questionnaire and depression symptoms by using Patient Health Questionnaire. Data entry and clearance were carried out by EpInfo version 7 and analysis was carried out by Statistical Package for Social Sciences version-20 software package. Data was examined using descriptive statistics and logistic regression, odds ratios and 95 % confidence intervals.

**Result:**

Over one third (38.3 %) of respondents met the symptoms criteria for depression. Gender, marital status, displaced previously as refugee, witnessing murderer of family or friend, lack of house or shelter and being exposed to increased number of cumulative traumatic events were significantly associated with depression among Somali refugees in Melkadida camp.

**Conclusion:**

The study revealed a relatively high prevalence of depression episode among refugees. Being female, divorced, deprived of shelter and witnessing the murder of family are most determinants of depression in refugees. Strengthening the clinical set up and establishing good referral linkage with mental health institutions is strongly recommended.

## Background

Psychological distress, psychosomatic complaints and clinical mental disorders such as depression and post-traumatic stress disorder are more prevalent among refugees than other populations [1].

Depression is a common mental disorder that presents with depressed mood, loss of interest or pleasure, decreased energy, feelings of guilt or low self-worth, disturbed sleep or appetite, and poor concentration. These problems can become chronic or recurrent and lead to substantial impairments in an individual’s ability to take care of his or her everyday responsibilities. At its most severe, depression can lead to suicide. Depression is the most common mental health outcome of exposure to war-related traumatic stressors [2].

Mental health problems affect society as a whole, and not just a small, isolated segment of the population. No group is immune to mental disorders but specifically the risk of depression is higher among the homeless, the unemployed, persons with low education, victims of violence, migrants and refugees, abused women and the neglected elderly [3].

Refugees are at high risk of psychiatric morbidity because of forced migration, traumatic events, and resettlement in unfamiliar environments [4]. Refugees and asylum seekers (those awaiting decisions on whether their refugee status is accepted) are at elevated risk of developing mental health problems as they have often experienced multiple and chronic adversities, and studies have demonstrated their higher prevalence of psychological disorders, in particular of depression and post-traumatic stress disorder [5].

Study among many refugees in Norway showed that refugees have been exposed to multiple traumatic events in their home countries, such as imprisonment in concentration camps, torture or multiple rapes that makes them to suffer from a variety of psychiatric problems for many years after resettlement [6].

Different study in the area of refugee has highlighted distress and psychiatric morbidity is known to be high in asylum seekers and refugees, with depression rates from 9.8 % to 67.4 % [6–8].

The study among Guatemalan refugees in Mexico showed that the lifetime prevalence of depression is 38.8 %. This study indicated that being women, being widowed, being married, witnessing to disappearances, witnessing murder of family or friend, experiencing 7 to 12 traumatic events and experiencing 13 to 16 traumatic events were associated with depression [9].

According to the cross-sectional study conducted with the tool of Hopkins Symptoms Checklist 25 (HSCL-25) among Karenina refugees living in the Thai-Burma border showed that the lifetime prevalence of depression is 41.8 % and refugees who had previous mental illness, those who had experienced a high number of traumatic events and women were at higher risk depression [10].

Studies conducted among refugee in Uganda and Southern Sudan identified high prevalence of depression which is 67.4 % and 49.9 % respectively [11, 12]. The prevalence of depression was significantly higher among Ethiopian immigrants and refugees who were exposed to pre-migration trauma, refugee camp internment, and post-migration stressful life events which is about 9.8 % [13].

Mental health problems affect society as a whole, and not just a small, isolated segment of the population. No group is immune to mental disorders but specifically the risk of depression is higher among the homeless, the unemployed, persons with low education, victims of violence, migrants and refugees, abused women and the neglected elderly [14].

The prevalence of depression among displaced people was reported higher in different parts of the world. Despite this, there is little study documented regarding mental health of refugees in Ethiopia. Likewise, the prevalence of depression among refugee is not studied in the nation particularly at Melkadida camp. Thus the study aims: to identify the prevalence and determinants of depression among refugees, and hence help for the early diagnosis and treatment of depression among refugee.

## Method

The study took place in May 2014 in Melkadida Southeastern part of Ethiopia and 890 Km far from Addis Ababa, capital of Ethiopia. The estimated population of the camp during the study period was 43, 380 people and all were Somali. Since November 2012,International Medical Corps (IMC) has been implementing community based mental health and psychosocial support services in Melkadida Camp which is integrating comprehensive clinical mental health and psychosocial services with health clinic in the camp .

Community based cross-sectional survey design was employed. The study populations were all adults aged 18 years and above living in Melkadida camp during the period. We excluded participants who are seriously ill and unable to communicate during the data collection time. The study used Patient Health Questionnaire (PHQ-9) as an outcome measure and Harvard trauma questionnaire (HTQ) which measures the level of exposure to trauma events (the finding to be presented elsewhere). Sample size was calculated using single population proportion formula by assuming the 95 % certainty and a maximum discrepancy of 5 % between the sample and the underlying population. Due to multistage sampling method used, a design effect of 2.0 was included which doubled the required sample size. The expected proportion of unusable questionnaires was set at 10 %.

Multistage probability sampling was used to select 831 households. At stage one from the twenty zones, seven zones were selected randomly using lottery method.

Then individual households in the chosen zones were selected using a systematic sampling technique after identifying an initial starting household by use of a random number. The sampling frame was the list of house number which was obtained from Melkadida camp administration and United Higher Commissioner for Refugee (UNHCR) local office. By this the interval (K) was calculated and every three house hold was visited. The sample size was distributed to each zone by proportional allocation. Adults in the selected household was further selected and interviewed. In the case of more than one eligible participant in the household, lottery method was used to select only one. For eligible participant who was not found at home, the interviewers had revisited the house hold three times at different time intervals and when interviewers failed to get the eligible participant, the household was registered as non-response.

Interviews were conducted with a randomly selected adult from each household at respondents’ homes. Semi structured questionnaire used to collect socio-demographic information, the displacement characteristics and substance use. Substance use measured as current users and ever users. Current users means when the respondent use specified substance (for non-medical purpose) in the last three months whereas ever users is when the respondent use specified substance (for non-medical purpose) even once in their life time.

A slightly adapted version of the original HTQ was used to identify exposure to trauma events [15]. It was not validated in the region and local language yet pretest was done to show its internal consistency. This consisted of 16 questions on lifetime exposure to traumatic events with a ‘yes/no’ response. Reliability coefficient Cronbach’s α in our study for the HTQ was 0.79 showing good internal consistency of the items.

Depression was measured by using PHQ-9 with a 3 point severity scale over the last 2 weeks. Based on the instrument standard PHQ-9 score ≥5 is considered as significant for meeting the symptoms of depression. This instrument was validated in Ethiopia. It incorporates Diagnostic and Statistical Manual for mental disorder version IV (DSM-IV) depression criteria with other leading depression symptoms into a brief self-report tool [15–17].

The data were collected by using pretested and interviewer administered questionnaire. The questionnaire was translated and delivered in Somali, the main language of Somali refugees at Melkadida camp. The translation had followed recommended guidelines, and involve forward and back translation.

A team of 6 data collectors were recruited for the survey. They all spoke fluent Somali and/or English and all had experience in data collection in Melkadida camp. Four days intensive training was provided for the data collectors on aims and intended value of the study; mental health and research, focusing on depression; review of the individual items in the survey; ethical issues of informed consent, confidentiality; and ensuring high interview quality and avoiding bias. Supervision and quality control were carried out by one supervisor and the principal investigator. Throughout the course of the data collection, interviewers were supervised at each site, regular meetings was held in between the data collection to discuss on problematic issues arising during interview and mistakes found during data collection was discussed and resolved.

The data collection took place from April 1st to May 30th 2014. Ethical approval was obtained from the Institutional Review Board of College of Medicine and Health Science, University of Gondar and supportive letter from Administration for Refugee and Returnee Affairs. Participants were informed about the aims and purpose of the study and its contribution to mental health services of the refugee. A signed consent form was used to ensure informed consent and clarify that no direct benefit could be expected from participating in the study. The full right was given to the study participants to refuse, stop and withdrawn from participation at any time during the data collection without loss of any entitlement. Confidentiality of respondents was maintained by using anonymous data collection tool.

Data were checked, coded and entered into Epi-info version 7 and exported to SPSS version 20 for analysis. Descriptive statistics was used to describe the study sample. Associations between risk factors and depression episodes were analyzed using bivariate and multivariate logistic regressions. The magnitude of the association between the different variables in relation to depression was measured using Odd Ratio (OR) and 95 % confidence interval (CI). Variables with *P* < 0.2 at 95 % confidence interval in bivariate logistic regression were entered into multivariate logistic regression to control the confounding effects. Variables with *P <*0.05 in multivariate logistic regression were considered as significantly associated with depression.

## Results

The survey included 847 individuals, among these 16 (13 questionnaires found to be incomplete and 3 individuals were not found at home) and excluded from the analysis registered as non-response.

### Socio demographic characteristics

831 interviews were completed with the overall response rate of 98.1 %. The socio-demographic data showed that about three quarter (74.6 %) of respondents were in the age between 18–40years with median age of 33, and 53.9 % were women and 46.1 % men. More than half (55.6 %) of the respondents were currently married. Majority of the study subjects (43.6 %) had never attended the school and only (8.9 %) had achieved college. Almost two third of the respondents (72.3 %) were not employed during the study period (Table 1).

**Table 1:** Socio demographic distribution respondents among Somali refugee at Melkadida camp, Southeast Ethiopia, 2014(n = 831)

Characteristics		Number	Percent
Age	18–40	620	74.6
	41–60	195	23.5
	>60	16	1.9
Sex	Male	383	46.1
	Female	448	53.9
Marital status	Married	462	55.6
	never married	132	15.9
	divorced	100	12
	widowed	66	7.9
	separated	71	8.5
Educational status	never attend the school	362	43.6
	primary school	395	47.5
	College	74	8.9
Employment status	employed	230	27.7
	not employed	601	72.3
Displacement characteristics	Never displaced previously	370	44.5
	Previously displaced as refugee	243	29.2
	Previously displaced as IDP	79	9.5
	Previously displaced as both refugee and IDP	48	5.8
	Displaced more than once	91	11

### Substance use status of study participants

About 30.7 % were current user and 33 % were ever user of khat. Out of this, male current user and ever user of khat were 22.7 % and 23.9 % respectively. Moreover, 1.9 % of the respondents reported current consumption of alcohol, and 2.9 % reported alcohol use at least once in their lifetime. About 23.7 % of the respondents were smoking tobacco currently (Table 2).

**Table 2:** Distribution of substance use among respondents of Melkadida camp, Southeast Ethiopia, 2014(n = 831)

Characteristics	Last three months		Life time	
	Frequency	Percent	Frequency	Percent
Cigarette use				
Users	195	23.5	197	23.7
Non users	636	76.5	634	76.3
Alcohol use				
Users	16	2.9	24	1.9
Non users	815	97.1	807	98.1
Chat use				
Users	255	30.7	274	33.0
Non users	576	69.3	557	67.0

### Exposure status to trauma events of study participants

Nearly one thirds (31.3 %) of respondents had ever experienced 8 or more of the 16 trauma events. Over half (57.4 %) of the respondents had ever experienced a combat situation. About 45.1 % had witnessed the murder of family or friends, and 53 % of respondents reported witnessing of unnatural death of family. Social deprivation is evidenced by the fact that three quarters (74.4 %) of respondents had ever lacked food or water, more than half (59.3 %) reported lack house or shelter and 44.4 % had been seriously ill without access to medical care.

Women generally reported higher rates of exposure to individual trauma events than men. Men have reported slightly higher exposure to 8 or more of the 16 trauma events included in the questionnaire (16.0 % of men compared to 15.3 % of women) (Table 3 and 4).

**Table 3:** The frequency distribution of exposure to traumatic event among Somali refugees at Melkadida camp, Southeast Ethiopia, 2014(n = 831)

Trauma events	Yes N (%)		No N (%)
	<12 month	≥12 months	
lack of food or water	264 (31.8)	354 (42.6)	213 (25.6)
Unnatural death of family	21 (2.5)	420 (50.5)	390 (46.9)
combat situation	45 (5.4)	432 (52)	354 (42.6)
Murder of family/friend	40 (4.8)	335 (40.3)	456 (54.9)
Very ill without medical care	98 (11.8)	271 (32.6)	462 (55.6)
Lack of housing or shelter	158 (19)	335 (40.3)	338 (40.7)
Being close to death	65 (7.8)	255 (30.7)	511 (61.5)
Forced separation from family	14 (1.7)	218 (26.2)	599 (72.1)
Tortured or beaten	28 (3.4)	246 (29.6)	557 (67)
Forced isolation from others	16 (1.9)	245 (29.5)	570 (68.6)
Murder of stranger	13 (1.6)	242 (29.1)	576 (69.3)
Forced to accept thoughts of other	78 (9.4)	334 (40.2)	419 (50.4)
Imprisonment	82 (9.9)	293 (35.3)	456 (54.9)
Being abducted or kidnapped	3 (0.4)	84 (10.1)	744 (89.5)
Rape or sexual abuse	2 (.2)	51 (6.2)	776 (93.4)

**Table 4:** The frequency distribution of cumulative exposure to traumatic event among Somali refugees at Melkadida camp, Southeast Ethiopia, 2014 (n = 831)

Cumulative trauma event recorded	Frequency	percentage
0–3 trauma event	230	27.7
4–7 trauma event	341	41.0
8–11 trauma event	207	24.9
12–16 trauma event	53	6.4

**Table 5:** Correlates of depression with socio-demographic characteristics, displacement characteristics and exposure to traumatic event among Somali refugee at Melkadida camp, Southeast Ethiopia, 2014 (n = 831)

Variables	Depression		Crude OR 95 % CI	AOR (95 % CI)
	Yes	No		
Sex				
Male	101	282	1	1
Female	187	261	2.0 (1.490, 2.686)	2.4 (1.748,3.363)^b^
Marital				
Married	146	316	1	1
Single	43	89	1.1 (.692, 1.581)	1.5 (.920, 2.290)
Divorced	50	50	2.2 (1.396, 3.355)	2.1 (1.313,3.423)^a^
Widowed	30	36	1.8 (1.069, 3.042)	1.6 (.933, 2.898)
Separated	19	52	.8 (.451, 1.386)	.8(.438, 1.462)
Displacement characteristics				
Never displaced previously	116	254	1	1
displaced as refugee	100	143	1.5 (1.093,2.145)	1.6 (1.069,2.278)^a^
Previously displaced as IDP	31	48	1.4 (.856,2.337)	1.2 (.687,2.138)
Previously displaced as both	12	36	.7 (.366, 1.454)	.9 (0.46,2.053)
Displaced more than once	29	62	1.0 (.626, 1.676)	.8 (.433,1.302)
Murder of family/friend				
Yes	175	200	2.7 (1.975,3.556)	2.8(1.631,3.172)^a^
No	111	339	1	1
Lack of housing or shelter				
Yes	193	300	1.7(1.221,2.217)	1.5 (1.05,2.07)^a^
No	95	243	1	1
Cumulative traumatic event				
0–3	61	169	1	1
4–7	90	251	.9 (.680, 1.451)	.7 (.179, 1.104)
8–11	107	100	2.9 (1.987, 4.423)	1.9 (1.245,3.176)^b^
12–16	30	23	3.6 (1.950, 6.698)	2.3 (1.131,4.585)^a^

Abbreviations: CI, confidence interval; COR Crude odds ratio, AOR adjusted odd ratio

^a^ Statistically significant variables (*P <* 0.01)

^b^ Statistically significant (*P* < 0.05)

### Prevalence of depression

According to the PHQ-9, 318 (38.3 % (95 % CI 34.9, 41.9)) adults were identified by PHQ-9 as having had a depressive episode in the two weeks preceding the survey.

### Factors associated with depression

Determinants which had significant associations on bivariate analysis at p-value ˂ 0.2 were then entered for multivariate analysis.

The following variables were associated (*p <*0.05) with increased odds of depressive symptoms:

Depressive symptomatology was associated with female gender, being divorced; forcefully displaced as refugee previously; witnessing murder of the family or friend and experiencing lack of house or shelter; experiencing 8 and more cumulative trauma events. Women were twice as likely as men to exhibit symptoms of depression (AOR = 2.4 CI (1.748, 3.363)) and the likelihood of having depression symptoms were higher in divorced (AOR = 2.1 CI (1.313, 3.423)).

Respondents who were forcefully displaced as refugee previously were more likely (AOR = 1.561 CI (1.069, 2.278)) to exhibit depression symptoms. In this study both Bivariate and multivariate analysis showed that there was no statistically significant association between depression and age category, religion, ethnicity, substance use and employment status.

Witnessing murder of the family or friend (AOR = 2.3; 95 % CI (1.631, 3.172)) and lack of house or shelter (AOR = 1.5; 95 % CI (1.05, 2.07)) showed significant associations with depression. The likelihood of having depression among those who had experienced 8–11 trauma event (AOR = 1.988 CI(1.145,3.176) and 12–16 trauma event (AOR = (2.3 CI (1.131,4.585) were higher. After adjusting for all variables the association of unnatural death of family and very ill without access to medical care with depression failed to resist (Table 5).

## Discussions

The overall prevalence of depression among Somali refugee was 38.3 %, not unlike prevalence rates found among Guatemalan refugees in Mexico and Karenina refugees living in the Thai-Burma border which 38.8 % and 41.8 % respectively [9, 10].

However, it was found to be higher than study conducted among Ethiopian immigrants and refugees in Toronto which was 9.8 % and among Vietnamese refugee in United States which was 20 % [13] [18]. The difference in this study and that of the Ethiopian immigrants and refugees in Toronto study could be as a result of more psycho social support, post migration growth than Somali refugee in Melkadida camp. The difference with that of Vietnamese refugee might be as a result of more pre and post migration stressful life events and environmental factors such as economic pressure among Somali refugee in Melkadida camp.

Our finding was lower than the prevalence of depression among Internally Displaced peoples (IDPs) and refugee in Southern Sudan and amongst internally displaced persons in northern Uganda which was 49.9 % and 67 % respectively [11, 12]. The higher prevalence among refugees in Southern Sudan and IDP in Uganda could be due to ongoing feeling of insecurity among refugees community and also might be due to difference in instrument.

The finding of a higher prevalence rate of depression among women than men in this study is similar to a pattern observed in different displaced population [9–12, 18–21]. However, this finding was opposite to the pattern observed in Ethiopian Immigrants and Refugees in Toronto [13]. The possible explanation for this pattern could be that the Ethiopian men may not be more willing to accept a drop in professional or social status, set aside educational goals, and assume dual responsibilities at work and at home. Moreover, women in this sample were more likely to have experienced conditions associated with depression (i.e. migration traumatic experiences and refugee camp internment) than men. Thus, Somali women compared with men are more likely to be exposed to adverse mental health consequences of migration and settlement stressors.

A number of significant associations of independent variables were found with outcomes of depression after adjusting for effects of other demographic and trauma exposure variables. Women are at particularly high risk of depression, as recorded in other studies on mental health of displaced populations [9, 10, 12, 18–21]. The elevated risk for depression could be explained by negative attitudes towards women, lack of acknowledgement for their work, fewer opportunities for them in education and employment, and greater risk of domestic violence. It also explained by hormonal changes during menstruation in women [22].

The finding reveals that being divorced was positively associated with depression. This finding was in line with study among Vietnamese refugee in United States which showed that being divorced is consistent risk factor for depression [18]. The finding implies the unstable marital relationship and the loss of partner increases the risk of having depression episodes.

Respondents who witnessed murder of family or friend were associated with depression. This study was consistent with the study conducted among Guatemalan refugees in Mexico [9]. In this study, being exposed to 8 or more out of 16 traumatic events was significantly associated with the outcome of depression. The dose–response relationship between exposure to traumatic events and depression is also similar with other studies of displaced population [3, 12, 19]. Exposure to multiple war stressors may lead to hopelessness and respondents who had experienced a high frequency of traumatic events were also more vulnerable to depression.

This study also showed that lack of basic social goods such as house or shelter had a significant association with outcomes of depression. This could be due to lacking basic needs like shelter make to have low self-esteem which leads to loss of interest in every activity and hopelessness.

There was strong association between forced displacement as refugee previously and depression. This was similar with the findings of the study conducted in Northern Uganda [12]. Possible reason could be repeated exposure to trauma event and confronting with adverse situations and ongoing stressors in different camp, which substantially impact their mental health.

So that, the finding of this study had a good argument back ground and evidence based to find out more or less similar prevalence rate compared to other community based studies.

### Limitation of the study

The possible limitations of this study were firstly, the study did not assessed the duration of stay in the camp which may have negative impact on the mental health of the refugee population. Secondly, the study was unable to consistently match the gender of interviewer and respondents. As a result, there may have been underreporting of certain sensitive traumatic events like rape. Thirdly, even though PHQ-9 was validated in Ethiopia with Amharic language, it was not validated with Somali language and also it is screening tool rather than diagnostic tool, thus the endpoint of depression is not certain.

Since the study design is cross-sectional study, it does not allow to infer causation.

Since these findings are based on a single lay interviewer administered questionnaire administration, not on a clinical appraisal, thus the endpoint of depression is not certain.

### Conclusions and recommendations

The study revealed a relatively high prevalence of depression episode among refugees. Being female, divorced, deprived of shelter and witnessing the murder of family are most determinants of depression in refugees. Strengthening the clinical set up and establishing good referral linkage with mental health institutions is strongly recommended, as it would lessen the detrimental effect of depression among the refugee population. Moreover, a meaningful resolution to the conflict in Somalia would facilitate a return of refugees back to their homes, support a healing process, and help refugees re-build their lives.
